# The Matrix protein M1 from influenza C virus induces tubular membrane invaginations in an *in vitro* cell membrane model

**DOI:** 10.1038/srep40801

**Published:** 2017-01-25

**Authors:** David Saletti, Jens Radzimanowski, Gregory Effantin, Daniel Midtvedt, Stéphanie Mangenot, Winfried Weissenhorn, Patricia Bassereau, Marta Bally

**Affiliations:** 1 Laboratoire Physico Chimie Curie, Institut Curie, PSL Research University, CNRS UMR168, 75005, Paris, France; 2 Sorbonne Universités, UPMC Univ Paris 06, 75005, Paris, France; 3 Univ. Grenoble Alpes, CEA, CNRS, Institut de Biologie Structurale (IBS), 71, avenue des Martyrs, 38000 Grenoble, France; 4 Department of Physics, Chalmers University of Technology, Gothenburg, Sweden

## Abstract

Matrix proteins from enveloped viruses play an important role in budding and stabilizing virus particles. In order to assess the role of the matrix protein M1 from influenza C virus (M1-C) in plasma membrane deformation, we have combined structural and *in vitro* reconstitution experiments with model membranes. We present the crystal structure of the N-terminal domain of M1-C and show by Small Angle X-Ray Scattering analysis that full-length M1-C folds into an elongated structure that associates laterally into ring-like or filamentous polymers. Using negatively charged giant unilamellar vesicles (GUVs), we demonstrate that M1-C full-length binds to and induces inward budding of membrane tubules with diameters that resemble the diameter of viruses. Membrane tubule formation requires the C-terminal domain of M1-C, corroborating its essential role for M1-C polymerization. Our results indicate that M1-C assembly on membranes constitutes the driving force for budding and suggest that M1-C plays a key role in facilitating viral egress.

Enveloped viruses exit the host cell via budding, where newly assembled virions become progressively wrapped by the membrane of the infected cells, before pinching off the cell surface by membrane fission. This process can be nucleated at the inner leaflet of the plasma membrane and requires the bending of the membrane into vesicles or “buds” deformed away from the protein-binding leaflet and exhibiting therefore a strong negative curvature^1^.

Influenza virus, member of the negative strand segmented RNA virus orthomyxoviridae family, is an example of a virus exiting his host by budding from the plasma membrane^2^. Influenza viruses are classified into the genera Influenza A, B and C where Influenza A and B are major pathogens^3^,^4^. Although influenza C is less widespread, it causes respiratory infections in young children^4^ and mild upper respiratory infections in adults, possibly due to the presence of protective antibodies acquired early in life^5^. All influenza viruses are pleomorphic, assuming either a spheroidal morphology with a diameter of ~100 nm or forming filaments with a length up to several micrometers and a diameter of ~100 nm^2^,^6^. Generally, laboratory-adapted strains of influenza virus A form mostly spherical particles and clinical isolates are often filamentous^7^,^8^. The membrane of influenza viruses contains the glycoproteins hemagglutinin (HA), neuraminidase (NA), the ion channel M2 while the M1 matrix protein forms a coat associated with the inner leaflet of the viral membrane. Viruses enter cells by endocytosis and exposure to the endosomal pH is required for membrane fusion leading to the delivery of the ribonucleoproteins (RNP) into the cytoplasm. For their egress, prior to virus budding, assembly of glycoproteins, RNPs and the matrix protein M1 is initiated at the plasma membrane in case of influenza A virus^2^. Upon completion of assembly, influenza A virus release depends on the action of M2^9^ in an ESCRT (Endosomal sorting Complex required for Transport) –independent process^10^,^11^.

Matrix proteins from negative strand RNA viruses constitute the major structural protein and are the driving force for budding^2^,^12^,^13^,^14^. However, influenza A M1 (M1-A) lacks a membrane targeting sequence and is recruited to the plasma membrane by HA and/or NA through interactions with their cytoplasmic tails^15^,^16^ or by the M2 protein^17^. Accordingly, heterologous M1 expression in eukaryotic cells does not lead to virus-like particle (VLP) formation although M1-VLP release was reported upon influenza A virus M1 expression with vaccinia virus and baculoviruses expression systems^18^,^19^. Nevertheless, fusion of a N-terminal membrane targeting sequence to M1 from influenza A virus can lead to VLP formation^17^ suggesting that M1 is sufficient for VLP formation once targeted to membranes. Due to the lack of efficient membrane targeting by influenza A virus M1 alone, co-expression of HA, NA, M2 and M1^20^ or of either HA or NA together with M1 and M2^21^ is otherwise required for release of VLPs. Furthermore, expression of HA or NA alone also leads to spherical^20^ and filamentous VLPs, respectively^21^. However, in the context of infectious virions, the spherical or filamentous morphology depends on M1 in the case of influenza A and C viruses^6^,^22^,^23^,^24^,^25^. M1 from influenza C virus (M1-C) has been shown to be the key component in inducing the formation of protrusions, called cord-like structures (CLS), emanating out of infected cells^26^ and in regulating their growth^23^,^27^ thus demonstrating a role for M1-C in membrane remodeling.

M1 from influenza A virus is composed of an N-terminal helical domain^28^,^29^,^30^,^31^ which carries positive charges implicated in membrane binding *in vitro*^32^. In addition, positively charged residues of M1-A (R76/77/78) are required for M1 membrane targeting and attachment as well as incorporation into virions^33^,^34^. However, the basic surface may not suffice for membrane interaction *in vivo*^35^. The N-terminal domain is followed by a short linker that was proposed to contain a zinc binding site^36^ and the C-terminal domain. The latter dimerizes *in vitro*^37^, is essential for M1 polymerization^38^ and provides a link to the ribonucleoprotein (RNP)^32^. Full-length M1 from influenza virus A forms an elongated structure^39^ containing a compact N-terminal domain and an extended and partially flexible C-terminal domain under low pH conditions^40^. Influenza virus A M1 polymerization is enhanced upon membrane binding^41^ and cryoelectron microscopy analyses suggest that M1 forms a helical polymer underneath the viral membrane^42^. Finally, during influenza A virus entry, M1 release from the viral membrane is induced by the low pH in endosomes in a two-step mechanism^43^, which may lead to structural changes in M1 before release^44^,^45^.

In the context of understanding cell-membrane-related processes and membrane biophysics, cell membrane mimics have attracted considerable interest^46^,^47^,^48^,^49^. Giant unilamellar vesicles (bilayers in the form of spherical vesicles with a diameter ranging from 10 to 100 μm, GUV^50^) in particular, are well-suited to investigate membrane deformation processes induced by colloids^51^, polymers^52^,^53^, viral proteins^54^, or toxins^55^ but also to probe membrane fission^9^,^56^.

Here, we combine structural studies and GUV-based experiments to understand the M1-C induced membrane deformation. We show that the N-terminal domain of M1-C adopts a similar fold as M1-A, despite the low sequence homology of both proteins, but coordinates two Mg^2+^ ions which increases the positive surface charge and may thus facilitate electrostatic interactions with negatively charged membranes. Full length M1-C adopts an elongated structure that tends to polymerize into ring-like or filamentous structures via lateral interactions of M1-C protomers. M1-C interaction with GUVs containing negatively charged lipids leads to inward membrane tubulation, topologically similar to virus budding. Although the N-terminal domain on its own interacts with GUVs, tubulation requires the C-terminal domain. Our study thus confirms that M1-C assembly on membranes constitutes the driving force for influenza C virus bud formation.

## Results

### Structure of M1-C

**Figure 1:**
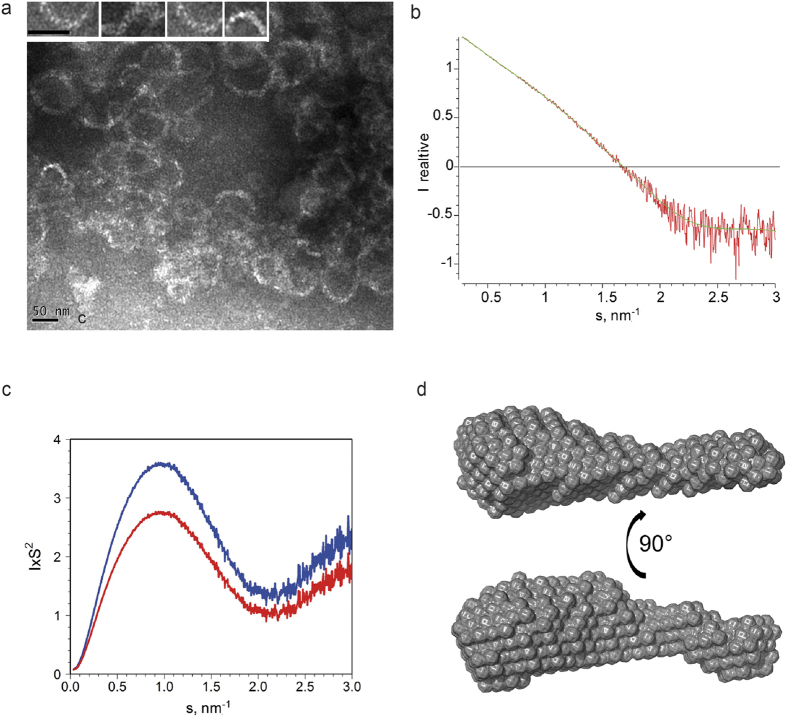
Structural analysis of M1-C. (**a**) M1-C forms polymers *in vitro* at low pH conditions as shown by negative staining electron microscopy. The width of the circular or spiral filaments is approximately 10 nm. The inset shows a close-up of some rod-like structures that associate laterally to form the filament. The scale bars are 50 nm. (**b**) M1-C also forms monomers at low pH conditions that produced the experimental SAXS data (red); the scattering pattern computed from the Dammin model shown in d is drawn in green. (**c**) Kratky plots for M1-C (red) and M1-A (blue). (**d**) Structural model of M1-C produced at low pH and reconstructed *ab initio.*

We expressed and purified full-length M1-C (Ann Arbor/1/1950), which produces monomeric M1-C as determined by size exclusion chromatography (SEC) and small angle x-ray scattering (SAXS) measurements (Table 1). In addition to the fraction of M1-C eluting as a monomer from the SEC column, ring-like and/or spiral structures were present in the void volume fraction of the SEC column at low pH conditions. The filaments have a width of approximately 12.5 + /−2.5 nm and ring diameters of approximately 50–100 nm (Fig. 1a). M1-C SAXS analysis (Fig. 1b) revealed a radius of gyration (Rg) of 2.85 nm and maximal dimensions of 11 nm in a low pH (pH 5.7) buffer. Although M1-C is also soluble at physiological pH, the SAXS data could not be analyzed due to a slight tendency to form higher order oligomers. M1-C (Ann Arbor/1/1950) and M1-A (Puerto Rico/8/1934 H1N1) share 14.29% sequence similarity (see Supplementary Fig. S1) and comparison of the SAXS data obtained at low pH suggests that they adopt similar structures. M1-A produces an Rg of 3 nm and maximal dimensions of 11 nm (Table 1) similar to previous analyses^39^,^40^. The molecular weight (MW) of the solute obtained from the extrapolated *I(0*) value was 27 kDa (27.9 kDa, calculated MW) for M1-A and 23 kDa for M1-C (26.9 kDa, calculated MW) confirming the monomeric state of M1 at low pH. The Kratky plots of M1-C and M1-A are bell-shaped, typical for folded proteins. At higher angles, elevated scattering is observed which indicates that part of the protein may be flexible (Fig. 1c). Ab initio modeling of M1-C produced an elongated 10 nm long structure fitting the corresponding experimental data with a discrepancy χ value of 0.916 (Fig. 1d) similar to the model calculated for M1-A^40^.

**Figure 2:**
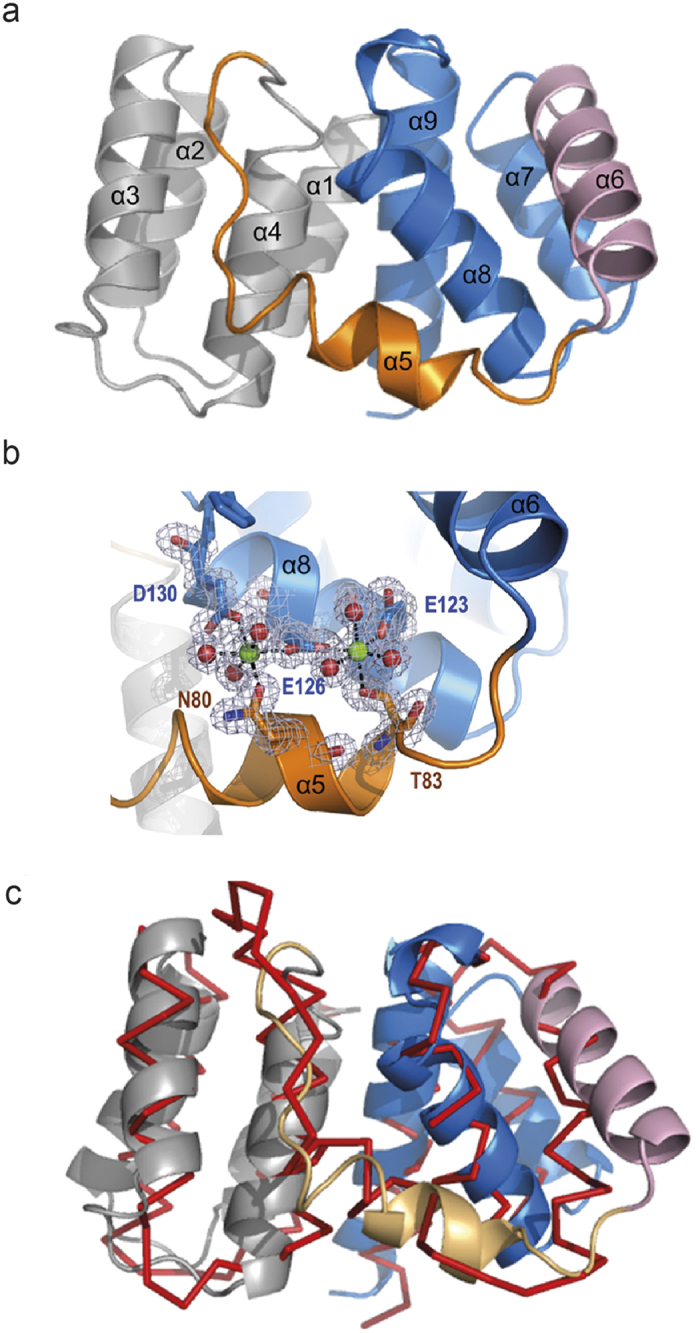
Structure of the M1-C N-terminal domain. (**a**) Ribbon diagram of M1-C composed of two four-helical bundles connected by helix 5. Alpha helices are labeled. (**b**) Close up of the Mg^2+^ binding sites. M1-C binds two Mg^2+^ ions coordinated by residues from helices 5 and 8 and six water molecules. Alpha helices are labeled. (**c**) Superposing of the Cα atoms of M1-A and M1-C reveals an overall similar fold and the displacement of helix 6.

**Figure 3:**
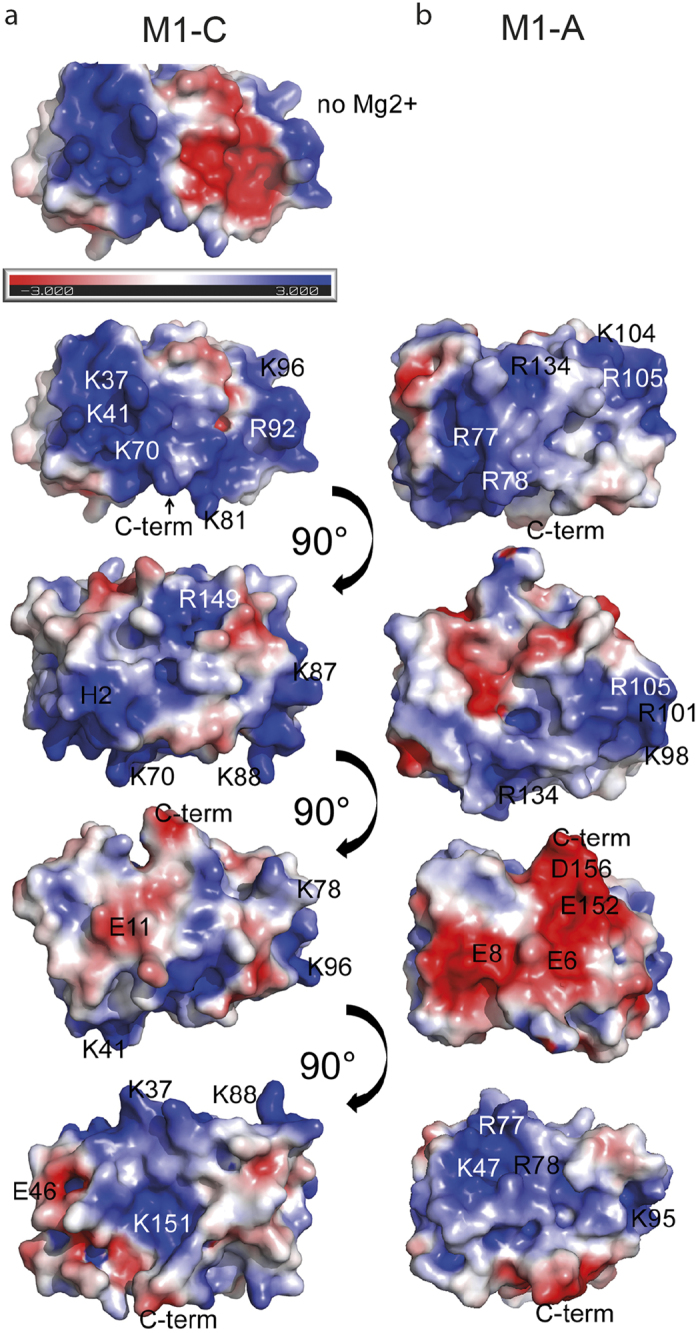
Electrostatic potential map comparison of M1-C and M1-A. M1 has large basic surfaces. Electrostatic potential map of M1-C (**a**) compared to the M1-A map (**b**). The electrostatic potential was calculated from −3.000 K_b_T/e_c_ (red) to +3.000 K_b_T/e_c_ (blue).

We solved the crystal structure of C-terminally truncated M1-C, (NTD, residues 1–155) by single anomalous dispersion (SAD) techniques to a resolution of 1.5 Å (Table 2). The structure is composed of a tandem repeat of two four-helical bundles (helices 1–4 and 6–9) that are connected by a long linker containing helix 5 (Fig. 2a). Interactions between the two four helical bundles and with the linker are hydrophobic as in the case of M1-A structures^28^,^29^,^31^,^39^. The overall fold is similar to known M1-A NTD crystal structures^28^,^29^,^31^,^39^, albeit their low sequence homology (see Supplementary Fig. S1). Superposition of the Cα atoms with M1-A (pdb 1EA3) reveals an overall r.m.s.d. of 2.86 Å. Major changes are observed for the position of helix 6, which moves approximately 10 Å outwards (Fig. 2c). A notable difference between the M1-A and M1-C structures is the unexpected coordination of 2 Mg^2+^ ions by helix 5 residues. The first Mg^2+^ ion is coordinated by Asn80, Asp130, Glu126 and three water molecules and the second Mg^2+^ ion interacts with Thr83, Glu123, Glu126 and three water molecules (Fig. 2b). Nevertheless the presence of Mg^2+^ may not have a structural role since circular dichroism showed no difference in melting temperature (TM = 62 °C) in the presence or absence of EDTA (ethylenediaminetetraacetic acid). However, the presence of the two Mg^2+^ ions changes the electrostatic potential of the M1-C surface. In the absence of Mg^2+^ this surface is substantially less positively charged (Fig. 3a, panel 1), while in the presence of Mg^2+^, the basic charge distribution is similar to that of M1-A (Fig. 3a, panel 1, and b). The other M1 surfaces carry a similar charge distribution, although the surface opposite to the basic surface (Fig. 3a,b; panels 3) is less negatively charged in the case of M1-C. It is thus very likely that M1-C employs also one of its basic surfaces to interact with negatively charged phospholipids present in the plasma membrane to initiate budding as suggested for M1-A^28^,^29^,^31^,^39^,^57^.

### M1-C binds to GUVs containing negatively charged lipids and induces inward tubulations

The interaction between M1-C and lipid membranes was investigated using GUVs of two different lipid compositions. The first composition contained 33 mol% of the negatively charged lipid DOPS, in addition to DOPE (33 mol%) and DOPC (33 mol%) (herein referred to as DOPS-GUV). The second composition consisted of lipids extracted from a natural tissue (porcine brain extract), which contains approximately 10 wt% PS lipids, to which 5 mol% PI(4,5)P2 was added (herein referred to as TBE-GUVs). Therefore, in this study, we worked with two extreme cases: a very simple composition, i.e. a “minimal model of the cell membrane” consisting of a protein-binding lipid and background non-interacting lipids and a more complex, native-like membrane, possibly reflecting the properties of native membranes more closely.

**Figure 4:**
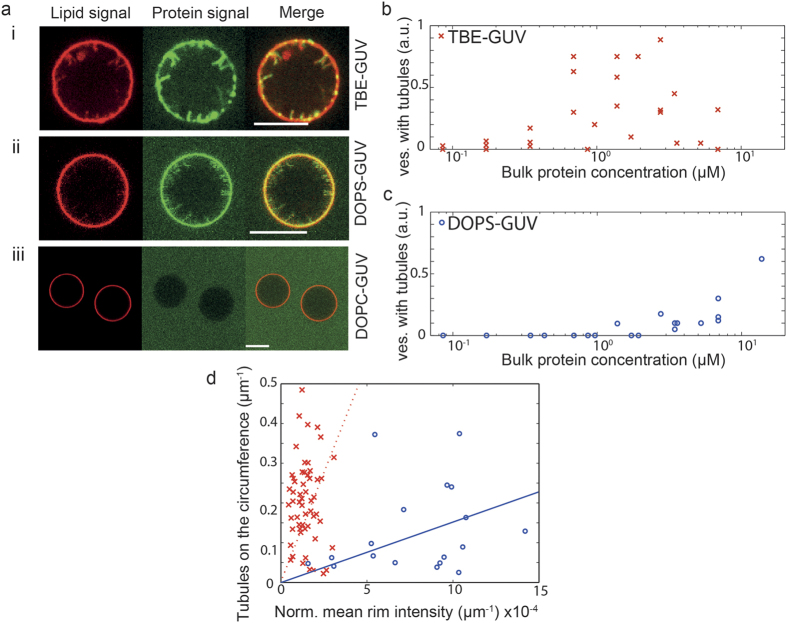
M1-induced tubulations. (**a**) Representative confocal images at the vesicles equator of (i) TBE-GUVs, (ii) DOPS-GUVs and (ii) DOPC-GUVs after incubation with M1. The red signal corresponds to bodipy-ceramide lipids incorporated into the GUV membrane and the green signal to Alexa-488 M1-C. Protein-induced membrane tubules are visible for the negatively charged vesicles of both compositions. No binding is observed in absence of negatively charged lipids. The protein concentration was 1.9 μM for the TBE-GUV experiment and 2.8 μM for the DOPS-GUVs and the DOPC-GUVs. Scale bars: 10 μm. The fraction of vesicles with tubulation in independent experiments for (**b**) TBE-GUVs and (**c**) DOPS-GUVs as a function of protein bulk concentration. (**d**) Tubule density at the equator as a function of the average intensity of the vesicle rim for TBE-GUVs (crosses) and DOPS-GUVs (circles) together with their respective linear fits (dotted line: TBE-GUVs; full line: DOPS-GUVs). Each data point corresponds to one analyzed vesicle and only vesicles with at least one tubule at the equator were taken into account.

After growing the GUVs by electroformation, proteins were added outside the GUVs at different bulk concentrations and fluorescence images were acquired at the equatorial plane of the vesicle. As visible in Fig. 4a M1-C interacted with vesicles of both compositions. The negatively charged lipids were a prerequisite for binding, as suggested by control experiments performed using vesicles containing DOPC only (DOPC-GUV). This observation is in good agreement with previous reports on M1-A^58^ further confirming that electrostatics play a key role in mediating membrane interactions for the M1 of both genera. Furthermore, this is in line with the general idea that both proteins exhibit a similar structure-function relationship. Interestingly, the interaction was shown to induce tubular membrane invaginations visible both in the protein (green) and lipid (red) channel (Fig. 4a). All protrusions were oriented towards the inside of the GUV thus topologically mimicking virus budding. Tubules were not observed in control samples without proteins (data not shown). These observations indicate that the protein alone is capable of negative curvature generation without the contribution of any other viral or cellular proteins. For both charged compositions, the tubules had diameters below the resolution of the microscope. Nevertheless, we could estimate from the relative intensities of bodipy-ceramide at the equator of the GUVs and in the tubules^59^ (see materials and methods for details) that the average tubule diameters range between approximately 120 and 250 nm; tubules generally appeared to be narrower at higher protein bulk concentrations M1-C (see Supplementary Fig. S2). The tubules were up to several micrometers long, appeared to be rather stiff and formed at M1-C concentrations above 3 μM for DOPS-GUVs and above 300 nM for TBE-GUVs. Tubulation was more common for the TBE-GUVs with >50% of the vesicles showing tubulation for protein concentrations in the low μM range (Fig. 4b). For the same concentration window, only approximately 10% of the DOPS-GUVs exhibited tubulation and the fraction of vesicles with tubules never exceeded 60% for all concentrations tested (Fig. 4b,c), in spite of the fact that DOPS-GUVs appeared to bind more M1-C, as shown by the evaluation of the mean intensity of the vesicle at the rim (Fig. 4d and Supplementary Fig. S3). We conclude that negative charges are required for M1-C membrane interaction and that membrane tubules budding into the GUV interior is more favorable for TBE-GUVs. To further investigate the possible relation between protein density and the tubulation process, the number of tubules on each vesicle was counted and normalized to the circumference of the vesicle equator, yielding the tubule density. As shown in Fig. 4d, the TBE-GUVs and DOPS-GUVs exhibited a clearly distinct tubulation behavior with higher tubule densities achieved at lower protein density for TBE-GUVs, indicating that less protein is required to deform the TBE-GUV membrane as compared to the DOPS-GUVs. For each individual composition, the dependence of the number of tubules per vesicle with the protein density was not very pronounced, although a weak linear trend can be found.

### M1-C clusters at the surface of the GUVs

**Figure 5:**
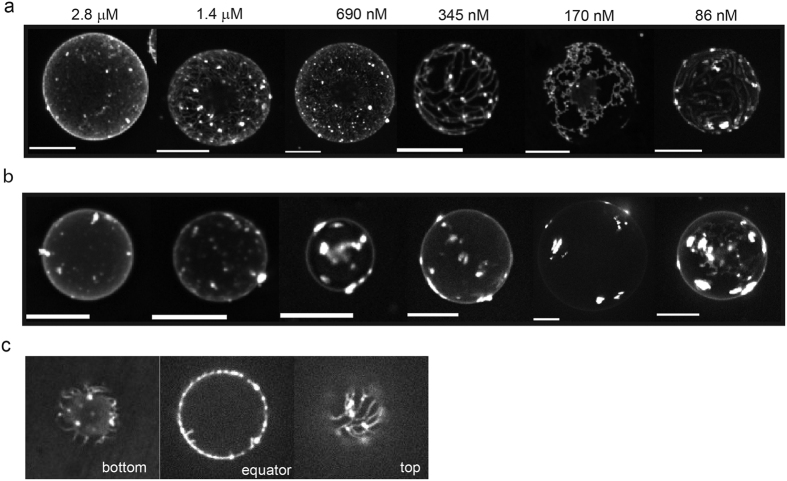
M1-C clustering at the GUV surface. Fluorescence projections of the protein signal on (**a**) TBE-GUVs and (**b**) DOPS-GUVs vesicles. For TBE-GUVs, proteins assemble into a network-like structure on the vesicle surface. For DOPS-GUVs the protein forms clusters but does not assemble into networks. A quantitative estimate of the number of clusters and network density can be found in Supplementary Fig. S4 (**c**) Z-stack images of a TBE-GUV vesicle incubated with 345 nM M1-C taken at the bottom (glass side) at the equator and at the top of the GUV. Scale bars: 10 μm.

**Figure 6:**
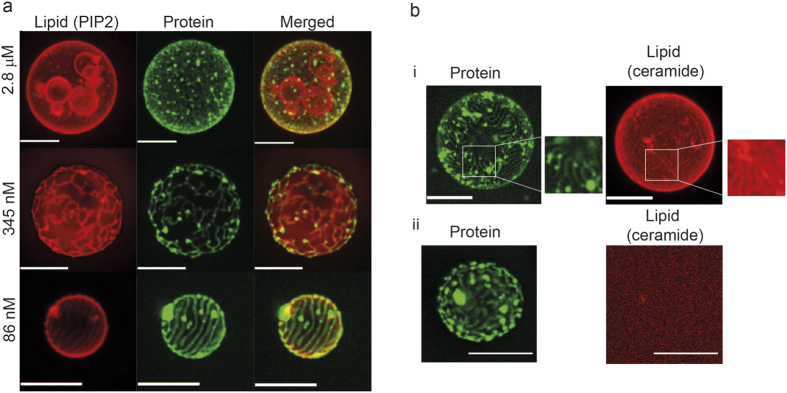
Colocalization experiment using labeled lipids. (**a**) Projections of TBE-GUVs with a negatively charged fluorescently-labeled lipid (Bodipy TMR PI(4,5)P2). The red signal corresponds to Bodipy TMR PI(4,5)P2 incorporated to the GUV membrane and the green signal to Alexa-488 M1-C. The negatively charged fluorescent lipids co-localize with the protein network. Scale bar: 10 μm. (**b**) (i) Projections of TBE-GUVs containing small amounts of bodipy-ceramide, a lipid that does not interact with M1 C. The red signal corresponds to bodipy-ceramide lipids incorporated to the GUV membrane and the green signal to Alexa-488 M1-C. In both cases, the protein bulk concentration was 1.4 μM. (ii) Control experiment using vesicles without fluorescent lipids at maximum laser power: only signal noise is detected, showing the absence of bleed-through between the fluorescence channels. Scale bar: 10 μm.

To gain more detailed insights into the assembly of the M1 protein layer on the lipid membranes, z-stacks were acquired by spinning disc confocal microscopy yielding a three-dimensional image of the GUVs. To visualize protein binding on the surface more easily, the stacks were projected into one plane, effectively portraying the vesicles either from below or above as shown in Fig. 5. In both cases, the M1 protein was found to cluster on the surface of the GUVs. For both vesicle types, binding appeared to be more homogeneous at high bulk protein concentrations, while the clusters became more prominent as the concentration was decreased. (For a quantitative analysis of cluster density see Supplementary Fig. S4) Interestingly, the cluster morphology was very distinct for both lipid compositions investigated. As visible in Fig. 5a, for TBE-GUVs, M1-C assembled into connected network-like structures. The projected representation used in Fig. 5 has the disadvantage that it becomes difficult to differentiate between structures on the surface of the GUV and tubules inside the vesicle, as tubules perpendicular to the imaging plane, for example, will appear as intense spots. However, careful inspection of the images revealed that the majority of the network structures visible in these projections were found at the GUV surface, as further illustrated in Fig. 6c and visualized in the Supplementary Movie. Such a network structure was never observed for the DOPS-GUVs; as visible in Fig. 5b, the protein rather assembles into more compact and round clusters.

To gain further insights into the process leading to protein clustering and tubulation, experiments were performed using low concentrations of fluorescently-labeled PI(4,5)P2 or fluorescently-labeled ceramide. Representative projections shown in Fig. 6a clearly display a colocalization of the fluorescent lipids (red) and fluorescent M1-C (green) indicating that both PIP(4,5)P2 and bodipy-ceramide are enriched at sites of M1-C interaction. This is further confirmed by estimating the Pearson correlation coefficient of our images and performing the statistical test described in ref. 60. We find that the colocalization lies above the confidence level of 3-times the standard deviation in all conditions (see Supplementary Table T1, for values and details). The observation of colocalization on bodipy-ceramide, a lipid that does not interact with M1-C (as shown in Fig. 4aiii) suggests that this enrichment originates, at least in part, from local membrane deformations at the vesicle surface. Indeed, the fluorescence increase reflects the surface projection of the curved membrane, thus a purely geometrical effect. Further analysis of the ratio of the lipid signal under the M1-C protein clusters and around them reveals that this effect dominates over local recruitment of PIP(4,5)P2 since the ratios are similar in both experiments with fluorescent labeled lipids (ratios: 1.75 ± 0.42 and 1,79 ± 0,32 for fluorescent PIP(4,5)P2 and for the fluorescent ceramide, respectively). These ratios further indicate that the deformation corresponds to less than half-cylinder with a diameter below optical resolution, since in this case the fluorescence would be locally increased by a factor π.

### The N-terminal domain is not sufficient to induce membrane tubulations

**Figure 7:**
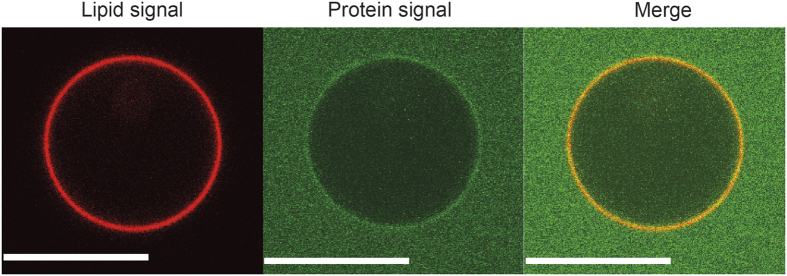
Membrane binding of the N-terminal domain of M1-C (M1C-NTD). Confocal images at the equator of TBE-GUVs after incubation with the M1C-NTD. The red signal corresponds to bodipy-ceramide lipids incorporated to the GUV membrane and the green signal to Alexa-488 M1-C. No tubulation was observed. The protein concentration was 2.8 μM. Scale bars: 10 μm.

To obtain further insight into M1-C function, experiments were performed using only the N-terminal domain of M1-C (M1C-NTD). M1C-NTD interacts with negatively charged membranes (Fig. 7), in agreement with the proposal that M1 NTD from M1-A contains the membrane binding region^32^,^39^. However, the N-terminal domain alone does not induce membrane deformation or tubulation (Fig. 7) and no protein clustering is observed, in contrast with full length M1-C. This strongly indicates that the C-terminal domain is required for M1-C polymerization possibly stabilizing interactions between protomers. Although M1 matrix protein assembly might drive membrane deformation, we cannot exclude the possibility that membrane deformation itself affects M1 assembly at the membrane.

## Discussion

The exact molecular mechanisms underlying influenza virus budding and the role of M1 polymerization in budding are still elusive. Here, we applied structural biology and a minimal membrane model system based on GUVs to elucidate the role of M1-C in membrane deformation and budding. We find that the structure of the N-terminal domain of M1-C resembles the structure of that of M1-A despite a low sequence similarity^28^,^29^,^31^,^39^. In addition, M1-C coordinates two Mg^2+^ ions, which are not present in the M1-A structure. Although Mg^2+^ coordination does not seem to have a structural role, its presence greatly enhances the positive charge of helices 5 and 8 on the N-terminal domain. Notably, the corresponding basic surface of M1-A was implicated in binding to negatively charged bilayers^28^,^29^,^31^,^32^,^39^, although electrostatic interactions may not constitute the sole driving force for membrane targeting^35^. In particular, helix 6 has a number of basic residues in M1-A and M1-C and mutations within helix 6 of M1-A affect membrane interaction^57^ as well as virus morphology^61^. This is consistent with the finding that membrane binding enhances M1 oligomerization and thus polymerization^41^ indicating that both membrane interaction and polymerization are concomitant processes. We also confirm that full length M1-C forms an elongated structure at low pH with an extended possibly flexible C-terminal domain, as predicted for M1-A by SAXS analysis^39^,^40^. We show that the C-terminal domain is required for M1-C polymerization into ring-like or filamentous structures *in vitro,* consistent with the role of conserved C-terminal residues in M1-A oligomerization^38^,^62^. Because we show that the M1C-NTD alone binds to GUVs but does not induce membrane tubule formation in our experiments with GUVs, we conclude that M1-C polymerization on membranes requires the C-terminal domain and generates M1 assemblies that are capable to reshape membranes. Because i*n vivo* imaging of M1-A polymers in native virions indicates the presence of a helical matrix layer^42^, a similar M1-C layer is expected to form during M1-C membrane tubule formation on GUVs. Furthermore, imaging of M1-A after release from virion membranes revealed spiral structures with a filament width of approximately 10 nm^58^. The width of these spiral filamentous structures is similar to the width of the M1-C polymers described here. Because the length of the M1-A and M1-C monomer structures is approximately 11 nm based on SAXS analyses, we suggests that elongated M1 structures line up via lateral interactions between neighboring N-terminal and C-terminal domains such as they expose the basic surface required for membrane interaction on the outside of the ring or spiral structures. We hypothesize that the negative curvature, inherent to the M1-C filaments, bends membranes upon filament growth into a helical M1 polymer^42^.

Our study on a model membrane system demonstrates that M1-C alone is capable of deforming a lipid membrane into tubular protrusions with negative curvature. The M1-C generated tubules are relatively stiff and up to several micrometers long. This morphology is reminiscent of that of filamentous Influenza C virions, but in contrast with the *in vivo* situation, M1-C-induced membrane tubules grow *in vitro* without restriction in the absence of the RNPs and/or the glycoproteins HA, NA and the M2 protein that is required for scission^9^ and thus restricts tubule length. Interestingly, the *in vitro* tubules exhibit diameters of the order of approximately 100–250 nm at high M1-C density, which resembles the diameter of influenza virions. Membrane deformation by viral matrix proteins has been shown so far for the vesicular stomatitis virus matrix protein that generates large (>1 μm) spherical buds that stay attached to the GUV membrane^54^. In addition, Ebola virus and New Castle disease virus matrix proteins also induce budding and release large (>1 μm) spherical vesicles from GUVs^63^,^56^. Because of the large size of the released vesicles, the physiological relevance may be limited. In contrast, our M1-C-induced membrane tubules are much smaller in diameter. Because the structure of the polymer is likely crucial to define the bud size, it is interesting to note that the M1-C helical or ring-like filaments imaged *in vitro* in solution by electron microscopy resemble the diameter of infectious virions. Furthermore, M1-C cluster formation upon binding to the lipid membrane is in agreement with the observation that M1-A also assembles elongated clusters with a network-like morphology or round-shaped clusters on supported lipid bilayers depending on the lipid composition^41^, although supported bilayers that prevent membrane deformation may not be ideal to study M1 polymerization.

In the case of TBE-GUVs, the presence of a network structure is correlated with tubulation. Linear assemblies and networks with very small mesh size (of the order of tens of nanometers) preceding membrane tubulation have been predicted by numerical simulations for proteins with an anisotropic shape, such as BAR domain proteins^64^,^65^,^66^, thus for systems where protein-protein interactions were absent, i.e. very different from the M1-C case. Currently, to the best of our knowledge, no simulation or experimental data on network assembly has been reported for polymerizing proteins such as M1-C. Therefore, it remains to be elucidated whether the network-like morphology represents a precursor state in the tubulation process or whether an alternative dead-end polymerization state, not relevant for the tubulation process. Furthermore, although it is likely that protein clustering and tubulation are related processes, as further suggested by the results with M1-NTD where no clustering or tubulation is observed, it is important to note that there is no direct correlation between size and amount of clusters and the amount of tubules on the GUVs.

We further show that the lipid composition affects M1-C membrane tubule formation. Tubulation was observed for the DOPS-GUVs, although much less pronounced than for the TBE-GUV mixture, where tubules were nucleated at lower M1 surface densities as compared to the DOPS-GUV system. This observed difference might have a number of origins related to the physical properties of the membrane (e.g. bending rigidity, spontaneous curvature, membrane tension or overall charge) or to the nature of the interaction with the negatively charged lipids. In the context of this work, membrane rigidity can be excluded as a likely cause for the difference in tubulation behavior observed for the two membrane compositions. Indeed, the bending rigidity for the DOPS-GUV was found to be approximately four times lower than the bending rigidity of the TBE-GUVs (12 ± 4K_B_T^59^ vs 46 ± 4.5K_B_T^67^) indicating that from a membrane-mechanics perspective, membrane deformation should be more favorable for the DOPS-GUVs (see for instance ref. 68). Another factor which may have a major influence on tubulation onset is the presence of lipids with an intrinsic negative curvature, such as Phosphatidylethanolamine (PE)-lipids^68^. Although DOPS-GUVs contain 33% DOPE while the TBE-GUVs contain only ~17% of PE-lipids, it is important to mention that about 60% of the TBE lipid composition is not known, thus it is possible that other lipids than PE with a negative curvature are present in this lipid mixture and contribute to facilitate tubulation.

Another hypothesis is that PI(4,5)P2 lipids amplify membrane tubulation. Although they do not increase M1 binding to the membrane, they may induce an arrangement of the proteins more favorable for M1-C polymerization and thus membrane tubulation. We therefore propose that the presence of PI(4,5)P2 in the bilayer may increase M1-C filament assembly required for budding. A detailed investigation on the influence of different membrane components on the tubulation process will be the subject of future investigations.

The egress mechanism of Influenza has attracted particular attention after the recognition that the virus does not depend on the ESCRT machinery to pinch-off the plasma membrane^10^,^69^, but is catalyzed instead by the M2 ion channel protein as demonstrated *in vitro*^9^. Our model system of M1-C induced membrane tubules that stay connected to the outer GUV membrane provides an excellent system to study influenza virus scission under more quasi physiological conditions to test hypotheses such as to the role of lipid domain formation^70^,^71^ or of the plasma membrane lipid composition^72^.

## Materials and Methods

### Reagents

Porcine brain total lipid extract (TBE), 1,2-dioleoyl-sn-glycero-3-phospho-(1′-myo-inositol-4′,5′-bisphosphate) (PI(4,5)P2), 1,2-dioleoyl-sn-glycero-3-phosphocholine (DOPC), 1,2-dioleoyl-snglycero-3-phosphoethanolamine (DOPE) and 1,2-dioleoyl-sn-glycero-3-phospho-L-serine (DOPS) were purchased from avanti lipids. Fluorescent BODIPY-Texas Red Ceramide (bodipy-ceramide) was from molecular probes and the BODIPY-TMR-PI(4,5)P2 from Tebu-bio. All other chemicals were purchased from Sigma-Aldrich.

### Protein expression and purification

The M1 protein of Influenza (strain C/Ann Arbor/1/1950) was cloned into a pET21d vector using the NdeI and XhoI restriction sites. Expression was performed in BL21 RIL (DE3) cells for 18 h at 20 °C. Cells were lysed by sonication in lysis buffer containing 150 mM NaCl, 50 mM Tris pH 7.5 and 1 μg ml^−1^ lysozyme. The cell lysate was centrifuged at 20 000 g and 277 K. The pellet fraction containing the protein in inclusion bodies (IBs) was washed five times in lysis buffer without lysozyme but with 2.0% (v/v) TritonX-100 and then twice in lysis buffer without detergent.

The purified IBs were solubilized in 6 M guanidinium hydrochloride (GdnHCl) and then diluted with water to a final concentration of 4 M GdnHCl. Refolding of M1-C was performed by the rapid dilution method with 100 mM NaCl, 100 mM Tris pH 8.0 and 0.8 M l-arginine as the refolding buffer. The refolded protein was further dialyzed in 20 mM MES pH 5.7, 10 mM NaCl and loaded onto a heparin column. The protein was eluted by applying a salt gradient between 10 mM and 1 M NaCl. Fractions containing M1-C were concentrated and further purified on a S75 (100/300) size exclusion chromatography column equilibrated in 10 mM MES pH 5.7, 100 mM NaCl.

### Electron microscopy analysis

For negative staining, M1-C was adsorbed to the clean side of a carbon film on mica, stained with 1% sodium silicotungstate pH 7.0, attached to a 400-mesh copper grid and transferred into a JEOL1200 EX II operating at 100 kV. The images were taken on a 2.7 k by 2.7 k Gatan ORIUS CCD camera at a nominal magnification of x20000.

### Crystallization, data collection and structure determination

M1-C NTD was crystallized in 100 mM Tris pH8.0, 200 mM MgCl_2_ and 25% PEG3350 by mixing 1 μl of the reservoir to 1 μl of protein at 15 mg/ml. The crystals were cryoprotected in the same conditions but with addition of 20% glycerol and flash cooled in liquid N_2_ before data collection. Se-Met derivatized crystals of M1-C NTD grew under the same conditions. A data set from a Se-Met crystal was collected at a wavelength of 0.97 Å on ID29 at the European Synchrotron Radiation Facility (ESRF, Grenoble, France). Data were processed with the program XDS^73^ and the crystals belong to space group C121 (Table 1).

The structure was solved by the SAD method employing the data set collected at the peak wavelength (0.9791 Å). The phases were improved and initial model building was performed with the Phenix program suite^74^. Further model building was completed manually with Coot^75^ and the model was refined with REFMAC^76^ to an R_factor_ of 18.7% and R_free_ of 21.9%. The model contains residues 2 to 155 and 100% of the residues are within the most favored and allowed regions of a Ramachandran plot^77^. Molecular graphics figures were generated with PyMOL (W. Delano; http://www.pymol.org). Coordinates and structure factors have been deposited in the Protein Data Bank with accession ID 5M1M.

### SAXS analysis

SAXS experiments were performed at the BM29 BioSAXS beamline at the ESRF (Grenoble, France). The proteins were purified by size-exclusion chromatography before SAXS measurements. M1-C and M1-A were analyzed at low pH conditions (10 mM MES pH5.7, 100 mM NaCl). The buffer of the size-exclusion chromatography step was used as reference for background subtraction for the SAXS measurement. For each sample, four different concentrations ranging between 0.8 and 4.4 mg/ml were measured. Protein solution (60 *μ*l) for each sample (and buffer) was exposed to X-rays and scattering data were collected. Ten individual frames of 30 s were collected for every exposure using a Pilatus 1 M detector (Dectris). Individual frames were processed automatically and independently using the software BsxCUBE, yielding individual radially averaged curves of normalized intensity versus scattering angle s = (4πSINθ)/λ. Frames were combined, excluding any data points affected by aggregation induced by radiation damage, to give the average scattering curve for each measurement (measured before and after every sample and background subtracted). SAXS data were processed and analysis steps were performed using the ATSAS package^78^,^79^. The final merged scattering data were further evaluated using PRIMUS. At low angle, the isotropic scattering data can be expressed as the Guinier approximation, I_exp_(s) = *I*(0) exp(−s^2^R_g_^2^/3). The isotropic scattering intensity I(q) was transformed to the distance distribution function *P(r*) using the program GNOM, which was also used to calculate the particle maximum dimensions D_*max*_. The optimum value of D_*max*_ was found when the R_*g*_ obtained from the *P(r*) plot was equal to that obtained from the Guinier analysis. For *ab initio* modeling of the SAXS data, 20 sets of independent models were calculated using Dammin^80^ and then averaged and aligned using DAMAVER^81^.

### Protein labeling

M1-C containing an extra C-terminal cystein was labeled with Alexa Fluor 488 C5 maleimide (Invitrogen). The dye was diluted to 1 mM in DMSO. M1-C in 20 mM HEPES, 300 mM NaCl, pH 7,5 at 0.5 mg/ml was incubated for 15 min in fresh TCEP (*tris*(2-carboxyethyl)phosphine) (40 ug/ml) followed by addition of the dye with a 1:1 protein:label ratio. After incubation overnight at 4 °C, free label was removed by size exclusion chromatography.

### Giant unilamellar vesicle preparation

Two different lipid compositions were used to form the GUVs. One natural composition (referred herein as TBE-GUVs) consisting of (in molar %): 94% BTE, 5% PI(4,5)P2 and 1% BODIPY^®^Texas Red ceramide. The other composition consisted of (in molar %): 33.2% DOPC, 33.2% DOPE, 33.2% DOPS and 0.5% BODIPY^®^Texas Red Ceramide (DOPS-GUVs). For control experiments, vesicles consisting of 99.5% DOPC and 0.5% BODIPY^®^Texas Red Ceramide (DOPC-GUV) (in molar %) was used. For the lipid colocalization experiments the concentration of BODIPY-Texas Red Ceramide was lowered to 0.15% (in molar %) or exchanged for the negatively charged lipid BODIPY^®^TMR PI(4,5)P2 at 0.1%.

The TBE-GUVs were obtained by electroformation on platinum wires. The platinum wires, 0.5 mm in diameter (Goodfellow SARL, France), were mounted in an in-house made teflon chamber, which places the wires 3 mm apart. The lipids were dissolved in chloroform to a concentration of 3 mg/ml, premixed and deposited on the wires in small droplets to a total volume of 3 μl. The chamber and wires were then dried in vacuum for 30 min before being rehydrated in buffer containing 10 mM TRIS, 50 mM NaCl and 100 mM Sucrose (pH 7.5). The chamber was sealed with sigillum wax (Vitrex, Denmark), high vacuum grease (Dow Corning, USA) and two glass cover slips (Menzel-Gläser, Germany). A function generator was then connected to the wires with a sinusoidal wave of 500 Hz and 280 mV RMS-voltage.

The GUVS were left to grow overnight (12–15 hours) at 4 °C and extracted through gentle pipetting directly above the wires.

The control experiments with DOPC-GUVs, and some of DOPS-GUVs experiments were performed after growing the vesicles by electroformation from indium tin oxide (ITO). coated glass slides (Präzisionsglas & Optik GmbH, Germany). In this case, a lipid film was deposited by adding 10 μl of a lipid mix (0.5 mg/ml) on the two slides and dried in high vacuum for 1 hour. To get a more homogeneous incorporation of the charged lipids into the vesicles, the lipid mix and ITO-plates were heated to 60 °C for 5 min prior and after spreading of the lipid films as described elsewhere^59^. The slides were then mounted together, 1 mm apart and a sucrose solution (100 mM) was added to a chamber made with sigillum wax (Vitrex, Denmark) around the dried lipid films. A sinus wave with RMS-voltage of 1 V and frequency of 10 Hz was then applied over the ITO -plates for 30–45 min (DOPS-GUV) or 60–120 min (DOPC-GUV). The vesicles were then extracted by pipetting directly from the chamber. The tubulation or protein binding behavior was found to be the same, regardless of the electroformation method chosen.

### Protein binding experiments

Protein binding experiments were performed using observation chambers produced in house from coverslips and coated with β-casein by incubation for 15–30 min (5 mg/ml, 10 mM TRIS, 100 mM NaCl at pH 7.5) followed by rinsing with experiment buffer. Protein binding experiments were carried out in TRIS buffer (10 mM Tris, 50 mM NaCl at pH 7.5 and adjusted to the same osmolarity as the vesicle growth medium by adding glucose). The vesicles were injected into the observation chamber filled with protein solution at required concentration leading in a 4:1 protein:GUV volume ratio and incubated for at least 30 min before observation.

### Microscopy

The vesicles were imaged using a spinning disk system set up on inverted Nikon eclipse Ti-E at The BioImaging Cell and Tissue Core Facility of the Institut Curie (PICT-IBiSA), member of the France-BioImaging national research infrastructure. The images were recorded on a CoolSNAP HQ2 camera. Some images were also recorded with an EMCCD iXon 897 Andor camera. All data for quantification based on fluorescence intensity were taken on the same microscope with the same camera (CoolSNAP HQ2 camera), using the same settings. The only parameter that was changed between samples was the exposure time. This was necessary to allow imaging of both low intensity samples and high intensity samples with the same laser power without saturating the detector.

### Data analysis

The data analysis was done with ImageJ and Matlab. To calculate the mean binding intensity of the vesicles at the equatorial plane, the OvalProfile plugin for ImageJ was used. The background signal was measured close to the vesicle and subtracted from the mean intensity. The same microscope and laser settings were used for all images except for the exposure time, which was normalized and scaled to the same value according to established methods using the dark pixel intensity of the camera^82^. To better visualize the z-stacks, the standard deviation z-projection from the top or bottom plane of the vesicle to the vesicle equator plane was used. For the tubulation quantification, the number of vesicles that displayed tubulation and the number of tubules at the equatorial plane of the vesicle were counted manually.

A rough estimate of the tubule diameter was obtained using a model-independent method: it makes use of the fact that the fact that the mean fluorescence intensity of a tubule normalized with the mean fluorescence intensity of the GUV is proportional to the tubule radius, as described in detail in^59^. The fluorescent intensity of the tubules was measured manually by drawing a line along the tubules and taking the mean intensity along that line, followed by subtraction of the background signal. The tubule intensity was then normalized with the GUV intensity (measured as described above) and multiplied with a proportionality constant of 200 nm^59^.

## Additional Information

**How to cite this article**: Saletti, D. *et al*. The Matrix protein M1 from influenza C virus induces tubular membrane invaginations in an *in vitro* cell membrane model. *Sci. Rep.* **7**, 40801; doi: 10.1038/srep40801 (2017).

**Publisher's note:** Springer Nature remains neutral with regard to jurisdictional claims in published maps and institutional affiliations.

## Supplementary Material

Supplementary Information

Supporting Movie

## Figures and Tables

**Table 1:** Summary of SAXS analyses for M1 from influenza virus C (M1-C) and influenza virus A (M1-A).

	Rg (nm)	I0	sRg max (nm)	Dmax (nm)	MW from I0 (kDa)	MW from volume (kDa)	Scattering power	Vc	Qr	Mass (kDa)
M1-A	3.0	25.5	0.6–1.2	11	27	30	8.0	3.1	3.2	26
M1-C	2.85	24.8	0.6–1.2	11	23	26	8.3	3.0	3.1	25

**Table 2:** Crystallographic data collection and refinement statistics.

	M1-C SAD
Data collection	
Space group	C121
Cell dimensions	
*a, b, c* (Å)	66.398 26.841 77.224
Α, β, γ (°)	90.00 108.40 90.00
Wavelength	0.9791
Resolution (Å)	31.5–1.5 (1.58–1.5)
*R*_sym_	0.063 (0.527)
*I*/σ*I*	12.1 (2.4)
Completeness (%)	99.3 (99.3)
Multiplicity	3.7 (3.8)
Refinement	
Resolution (Å)	1.5
No. Reflections Test set reflections	20868 1074
*R*_work_/*R*_free_	18.8/21.9
No. atoms	
Protein	1235
Ligand/ion	2 Mg^2+^
Water	186
*B*-factors	
Protein	26.4
Ligand/ion	30
Water	36.1
R.m.s deviations	
Bond lengths (Å)	0.006
Bond angles (°)	0.969
