# Supplementary material for: The Matrix protein M1 from influenza C virus induces tubular membrane invaginations in an in vitro cell membrane model
Source: Sci Rep. 2017 Jan 25;7:40801. doi: 10.1038/srep40801 (PMC5264427; doi:10.1038/srep40801)
Supplement: Supplementary Information [file srep40801-s1.pdf]

Supplementary information

The Matrix protein M1 from influenza C virus induces tubular membrane invaginations in an *in vitro* cell membrane model

David Saletti[1,2*], Jens Radzimanowski[3*], Gregory Effantin[3], Daniel Midtvedt[4], Stéphanie Mangenot[1,2], Winfried Weissenhorn[3,+], Patricia Bassereau[1,2,+], and Marta Bally[1,2,4 +]

[1]. Laboratoire Physico Chimie Curie, Institut Curie, PSL Research University, CNRS UMR168, 75005, Paris, France
[2] Sorbonne Universités, UPMC Univ Paris 06, 75005, Paris, France
[3] Univ. Grenoble Alpes, CEA, CNRS, Institut de Biologie Structurale (IBS), 71, avenue des Martyrs, 38000 Grenoble, France
[4] Department of Physics, Chalmers University of Technology, Gothenburg, Sweden

*Contributed equally

+corresponding authors: winfried.weissenhorn@ibs.fr; patricia.bassereau@curie.fr; bally@chalmers.se

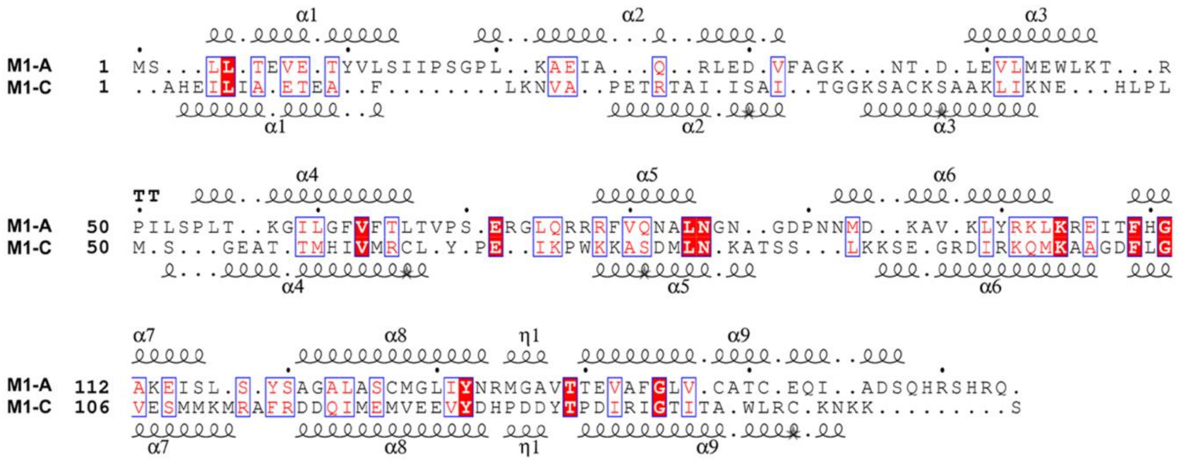

Figure S1. Sequence comparison of M1-A and M1-C. Sequence alignment of M1-C (Ann Arbor/1/1950) and M1-A (Puerto Rico/8/1934 H1N1) shows 14.29 % similarity. The secondary structure elements are shown above and below the primary sequences.

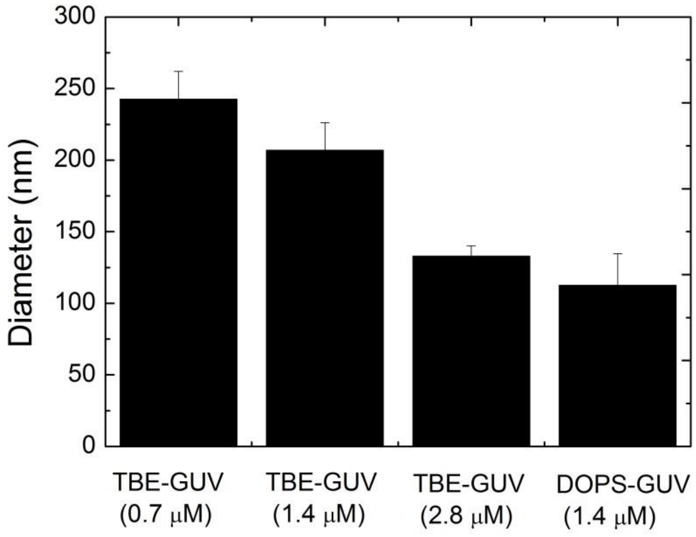

Figure S2. Tubule diameters estimated by fluorescence microscopy. The average tubule diameter was estimated from the relative intensities of the bodipy-ceramide signal at the GUV equator. The data is shown for both lipid compositions and at different protein concentrations (in brackets). Error bars: standard errors of mean. n=8-34.

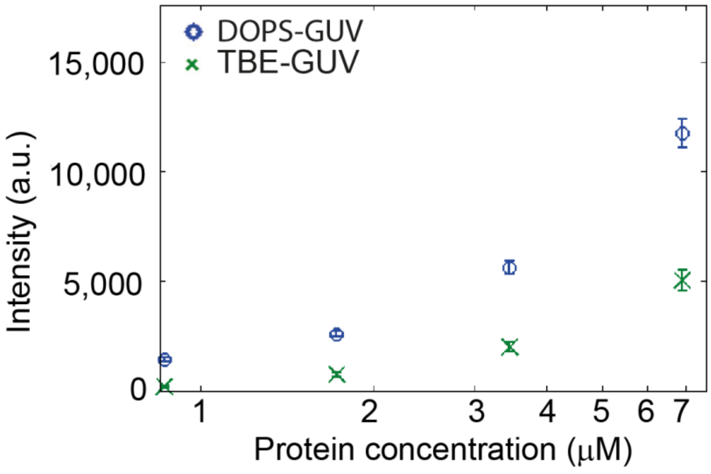

Figure S3. M1 binding as function of protein bulk concentration. Average fluorescence intensity of protein bound at the vesicle equatorial plane as function of the bulk protein concentration for TBE-GUVs (crosses) and DOPS-GUVs (circles) for a representative measurement. The measurements were all performed on the same microscope with identical settings except for the exposure times that were later scaled to the same value. The error bars represent the standard errors of mean. For all concentrations and in both graphs n=20-33.

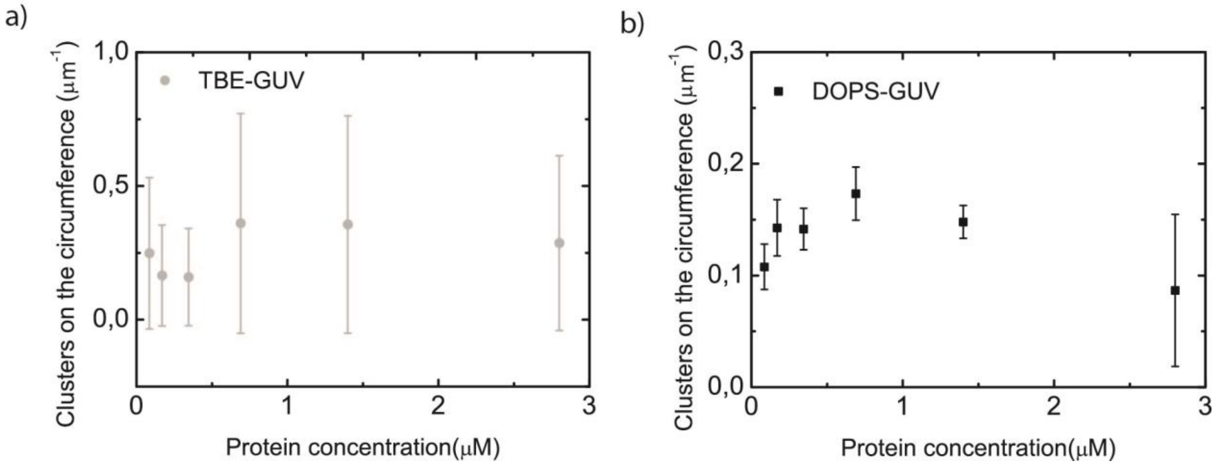

Figure S4. Analysis of the cluster density. The cluster density on (a) TBE-GUV and (b) DOPS-GUVs was analyzed by visual inspection of images at the equator and cluster counting. The obtained value therefore reflects the cluster surface density for round-shaped clusters (DOPS-GUVs) or the network density (TBE-GUVs). No dependence on the protein concentration could be resolved for the TBE-GUVs while the cluster density on DOPS-GUVs increases for protein concentrations up to 690 nM before decreasing. This is in line with the observation that at low protein concentration the amount of clusters detectable depends on the protein concentration while in the higher protein range, the protein coating becomes homogeneous leading to a decrease in the number of clusters counted. n>20 for all averages; error bars are the standard errors of mean.

Table T1. Semi-quantitative analysis of the colocalization events shown in Figure 6. We use the Pearson correlation coefficient as a quantitative measure for the degree of colocalization present under the different conditions. Since both species are localized to the surface of the vesicle, there is an inherent bias in the correlation coefficient when applied directly to the 3D intensity distributions. To correct for this, we project the 3D intensity distributions onto the surface of a sphere prior to calculating the correlation coefficient. To test the significance of the measured colocalization we employ the statistical test developed in Costes *et al.* (ref 59 in the main text). The last step was repeated 20 000 times for each image to obtain the expected statistical distribution of correlation coefficient for a non-colocalized sample. The significance of the obtained Pearson correlation coefficient was then determined on the basis of this distribution.

**Fluorescent lipid**	**Protein concentration**	**Pearson coefficient**	**Pearson coefficient (scrambled image)**
Bodipy TMR PI(4,5)P2	2.8 mM	0.42*	0.26±0.02
Bodipy TMR PI(4,5)P2	345 nM	0.31	0.06±0.03
Bodipy TMR PI(4,5)P2	86 nM	0.26	0.04±0.05
BODIPY-Texas Red Ceramide	1.4 mM	0.33	0.1±0.03

*The estimation of this coefficient is affected by the multilamellarity of the vesicle imaged which shows smaller vesicles enclosed in the outer GUV. See Figure 6 (top)

Supporting movie: Z-stack of a GUV acquired with spinning disc microscopy. Z-stack images of the TBE-GUV vesicle shown in Figure 6c and incubated with 345 nM M1-C.
